# Alar Battens Grafts Versus Lateral Crural Strut Grafts: A Systematic Review of Postoperative Outcomes

**DOI:** 10.1002/ohn.70010

**Published:** 2025-09-08

**Authors:** Jarrett M. Jackson, Nicole G. DeSisto, Rahul K. Sharma, Rachel Walden, Shiayin F. Yang, Scott J. Stephan, Priyesh N. Patel

**Affiliations:** ^1^ Division of Facial Plastic and Reconstructive Surgery, Department of Otolaryngology Vanderbilt University Medical Center Nashville Tennessee USA

**Keywords:** alar batten graft, lateral crural strut graft, nasal obstruction, rhinoplasty

## Abstract

**Objective:**

To systematically compare functional outcomes and postoperative cosmetic satisfaction following alar batten graft (ABG) versus lateral crural strut graft (LCSG) placement for patients with nasal valve incompetence.

**Data Sources:**

Pubmed and Embase searches (1995‐2025) with terms for nasal obstruction, LCSG, ABG, and functional/cosmetic outcomes.

**Review Methods:**

Relevant studies with documented preoperative nasal valve incompetence, confirmed surgical intervention with either ABG or LCSG, and their associated postoperative outcomes were identified. Review methods adhered to Preferred Reporting Items for Systematic Reviews and Meta‐analyses (PRISMA) guidelines. Due to heterogeneous study designs and incomplete variance data, the findings are presented as ranges without a quantitative meta‐analysis.

**Results:**

Fifteen studies (n = 1052 patients; LCSG = 690, ABG = 362) were included. Among eight LCSG studies, mean nasal obstruction symptom evaluation (NOSE) score improvements ranged from 15.8 to 65.6 points, and visual analog scale (VAS)‐function improvements ranged from 10.1 to 44.3 points. Seven ABG studies demonstrated NOSE improvements of 25.6 to 69.0 points, and VAS‐function improvements ranged from 24.0 to 54.5 points. Validated cosmetic patient‐reported outcome measures (Standardized Cosmesis and Health Nasal Outcomes Survey [SCHNOS]‐C, VAS‐cosmesis, and Facial Aesthetic Clinical Evaluation‐Questionnaire [FACE‐Q]) were used in LCSG cohorts, showing consistent esthetic gains. No ABG studies employed a standardized cosmetic measure. Heterogeneity precluded pooled analysis.

**Conclusion:**

Both ABG and LCSG yield substantial functional improvements for nasal valve incompetence across numerous studies. Cosmetic satisfaction appears favorable for LCSG but is undercharacterized for ABG due to a lack of reported standardized patient outcome measures. Large, prospective, and multi‐institutional studies investigating outcome differences are needed, given the significant heterogeneity across study methods in the existing literature.

Nasal obstruction causes significant quality of life impairment. It affects up to one‐third of the US population, with nearly two‐thirds of severe cases attributable in part to valve collapse.^1^, ^2^ The internal nasal valve (INV) is bound by the dorsal nasal septum medially, the caudal edge of the upper lateral cartilage laterally, and the head of the inferior turbinate posteriorly. It is the narrowest segment of the nasal airway and accounts for up to two‐thirds of total nasal airway resistance. Thus, dysfunction leads to both insufficiency and collapse. The external nasal valve (ENV) is the area within the nasal vestibule bordered by the alar rim, nasal sill, caudal septum, and lower lateral cartilages. The primary structural component of the ENV is the paired lower lateral cartilage, with any anatomical disruption of this area leading to nasal valve collapse and subsequent obstruction.^3^, ^4^, ^5^

There are several options for the surgical management of nasal valve collapse that have demonstrated excellent cosmetic and functional results. The lateral crural strut graft (LCSG) is frequently used to address insufficient support of the ENV, whereas the alar batten graft (ABG) is an additional surgical option for the correction of either external or INV collapse.^6^

First described by Toriumi in 1997, the ABG is a curvilinear cartilage graft placed at the point of maximal lateral wall collapse over the alar cartilage.^7^ ABG may be used to correct INV collapse due to inadequate strength of the lateral wall, frequently associated with previous reductive rhinoplasty. ABG may also provide improved structural support of the ENV, preventing valve collapse and improving symptoms of nasal obstruction. Functional improvement following placement of ABG has been well demonstrated in the literature.^7^, ^8^, ^9^ However, given the superficial placement of ABGs, they have previously been associated with increased fullness at the site of the graft, with several studies noting patient perception of fullness in the scroll region postoperatively.^7^, ^10^

The LCSG is a straight or slightly curved cartilage graft placed on the deep surface of the lateral crura, unlike the ABG, thereby requiring more meticulous dissection. LCSGs also successfully address weak or mispositioned lower lateral cartilage to reduce ENV dysfunction and collapse. Placement of LCSG has consistently resulted in improved functional and esthetic outcomes in both primary and revision rhinoplasty cases.^11^, ^12^ Much of recent literature has included objective and subjective outcomes following placement of ABG or LCSG for nasal valve collapse and obstruction. However, to our knowledge, no study has systematically compared the available literature on functional and cosmetic outcomes following either LCSG or ABG placement. We aim to assess currently available functional outcomes and postoperative cosmetic satisfaction rates for patients undergoing surgical placement of ABG or LCSG for nasal airway obstruction due to nasal valve incompetence.

## Methods

### Research Strategy

A systematic review of the literature was conducted in October 2022 and updated in May 2025 following the Preferred Reporting Items for Systematic Reviews and Meta‐analyses (PRISMA) guidelines. PubMed and Embase databases were systematically searched using Boolean combinations of the key search terms “Nasal Obstruction,” “Lateral Crural Strut Graft,” “Alar Batten Graft,” and “Functional/Cosmetic Outcomes” from the years 1995 to 2022, then re‐queried for October 2022 to May 2025 with additional terms (“cosmetic outcome,” “esthetic outcome,” and “outcome”) to capture updated esthetic measures. A complete accounting of the search criteria may be found in Supplemental Material S1, available online. A biomedical librarian (R. Walden, MLIS) assisted with search strategy development, and a biostatistician advised on data presentation. All references were uploaded to EndNote reference management software where duplicates were removed. The reference lists of all included studies were examined for additional relevant publications that may not have been produced in the original query. Following data collection, no quantitative meta‐analysis was performed due to the variability of the data reported across the studies.

### Study Selection

Inclusion criteria were prespecified according to the PICO framework: (1) population: adult patients undergoing functional nasal valve surgery (with or without concurrent cosmetic rhinoplasty) for nasal airway obstruction due to nasal valve incompetence; (2) intervention: surgical placement of ABGs; (3) comparison: LCSGs; and (4) outcome: nasal airway obstruction resolution, postoperative cosmetic satisfaction, and revision rates. Exclusion criteria included pediatric populations (age < 18), cadaveric studies, single case reports, “How I do it” articles, non‐English articles, and cleft rhinoplasty. Studies of interest were high‐quality retrospective/prospective studies, case series, and randomized control trials. These studies all used ABGs or LCSGs and had documented postoperative obstructive and cosmetic outcome measures (Facial Aesthetic Clinical Evaluation‐Questionnaire [FACE‐Q], Sino‐Nasal Outcomes Test (SNOT‐22), Standardized Cosmesis and Health Nasal Outcomes Survey [SCHNOS], nasal obstruction symptom evaluation [NOSE], and visual analog scale [VAS] surveys).

### Data Collection Process

Article abstracts and titles were independently screened by two reviewers (N.G.D. and J.M.J.). If there was a question about the content of the reference, it was also included for full‐text review. Disagreements were resolved via discussion between the two reviewers and input from an additional author (P.N.P.). Following initial title and abstract screening, full‐text review and data extraction were completed by the same independent reviewers. Scores on the NOSE and VAS scales were converted to be out of 100 to improve homogeneity between reported results. All authors approved the final data set.

### Data Items

The variables collected from each study included the following: author, publication year, graft type, primary versus revision, length of follow‐up, primary outcome measure (NOSE vs SCHNOS vs VAS), mean/median pre‐op score, mean/median post‐op score, and mean difference.

### Risk of Bias Assessment

The quality and risk of bias of the included studies were assessed using the Newcastle‐Ottawa Scale (Supplemental Material S2, available online).

## Results

**Figure 1:**
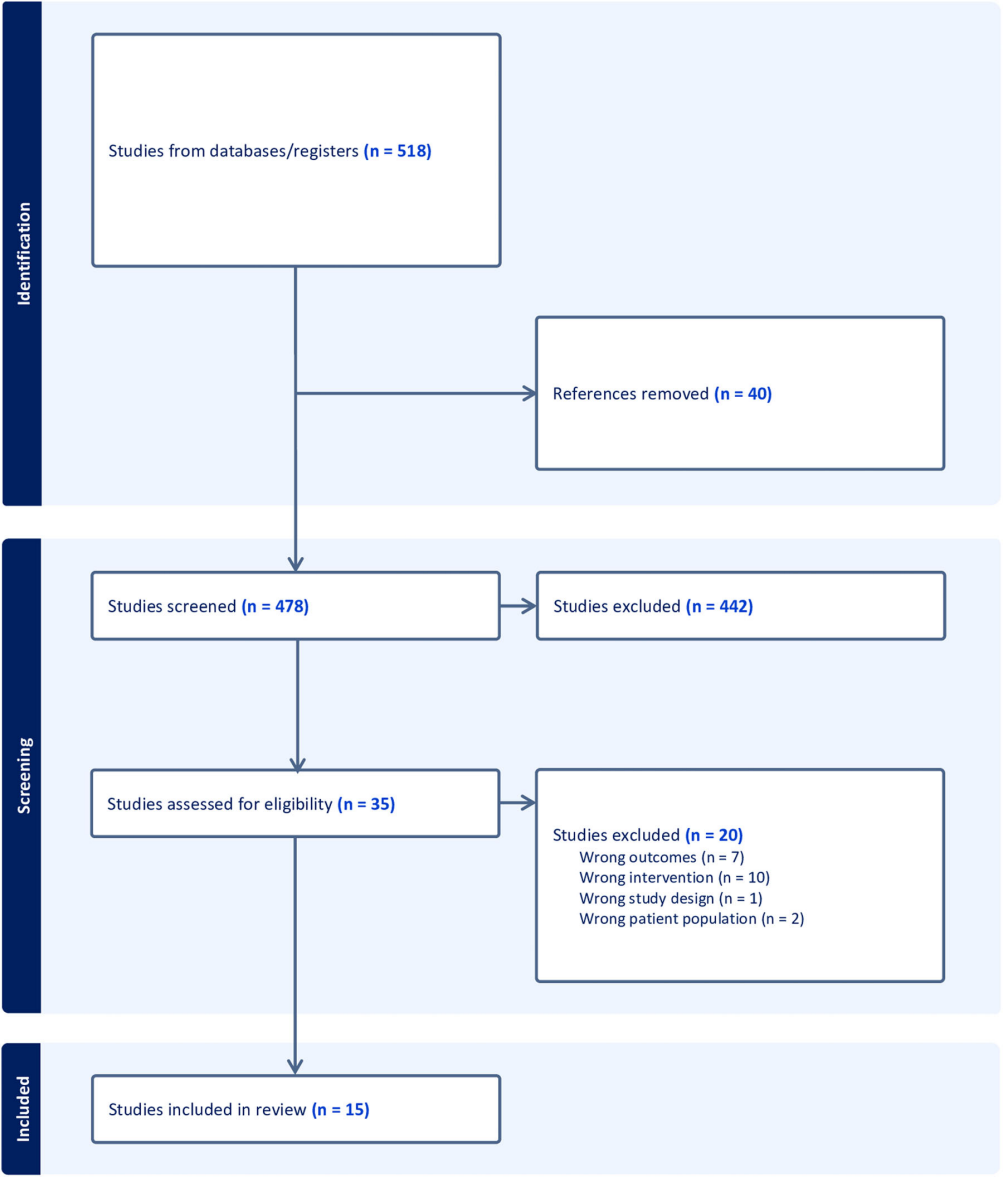
Preferred Reporting Items for a Systematic Reviews and Meta‐analyses flow diagram.

Our database search resulted in the identification of 518 unique articles uploaded to Covidence systematic review software (Veritas Health Innovation). Thirty‐one articles were included for full‐text review, with 15 being included for full data extraction and analysis (Figure 1). Across the 15 included studies, a total of 1052 patients underwent either alar batten (n = 362) or LCSG (n = 690) placement with documented postoperative outcomes. In studies lacking a clear subgroup N, the largest outcome‐specific sample size was used. Demographic and outcome details are summarized in Tables 1 and 2, respectively. Because of heterogeneity in study design, outcome instruments, and incomplete variance data, we present outcome data as ranges rather than pooled means and did not perform a quantitative meta‐analysis.

**Table 1:** Article Demographics Summary

	ABG	LCSG
Number of articles	7	8
Total N	362	690
Mean age reported	40 (range 18‐75)	33 (range 18‐52)
Rhinoplasty approach	Open	Open

Abbreviations: ABG, alar batten graft; LCSG, lateral crural strut graft.

**Table 2:** Article Outcomes Details

Author (year)	Graft type	Primary/revision	Follow‐up, mo	Outcome measure	N	Pre‐op mean/median*	SD	Post‐op mean/median*	SD2	Mean Δ	*P* value
Abdelhamid (2024)	LCSG	Revision	12	SCHNOS‐O	42	55	32.9	213.75	16	45.75 (32.49)	*P* < .0001
				SCHNOS‐C		69.24	26.97	13	21.2	55.67 (29.67)	*P* < .0001
				SCHNOS‐6		3.48	1.61	0.82	1.43	2.64 (2.23)	
				VAS‐function		59.5	28.5	14	14.6	44.3 (28.2)	*P* < .001
				VAS‐cosmesis		3.88	1.61	7.67	2.94	3.5 (4.02)	*P* = .0007
Toriumi (2023)	LCSG	Both	12‐24	NOSE	266	22.97	NR	7.22	NR	15.75	*P* < .0001
Hismi (2022)	LCSG	Primary	12	NOSE	141	70 [55‐83]*	NR	23 [12‐36]*	NR	42 ± 22.8	*P* < .001
				FACE‐Q (nasal)		50 [42‐69]*	NR	74 [59‐93]*	NR	16.7 ± 22.6	*P* < .001
				FACE‐Q (nostrils)		64 [34‐78]*	NR	84 [64‐100]*	NR	19.6 ± 25.5	*P* < .001
				FACE‐Q (social)		73 [58‐81]*	NR	80 [66‐96]*	NR	8.6 ± 15.5	*P* = .001
				NPIF (L/min)		60 [50‐90]*	NR	73 [55‐100]*	NR	10.7 ± 28.1	*P* = .001
Abdelwahab 2021	LCSG	Both	10	VAS‐function zone 1	7	20	22.31	10	10.4	11.9	*P* = .209
				NOSE zone 1	7	27.14	26.26	6.43	8.02	20.71	*P* = .067
				SCHNOS‐O zone 1	1	5	NR	15	12.2	10	NR
				VAS‐function zone 2	27	54.4	30.12	20.12	22.3	34.38	*P* < .001
				NOSE zone 2	31	58.87	30.57	21.93	19.4	36.94	*P* < .001
				SCHNOS‐O zone 2	5	65	41.83	2.22	16.2	62.78	*P* = .037
				VAS‐cosmesis	9	4.13	1.73	9.07	1.44	4.94	*P* < .001
				SCHNOS‐C	5	55.33	24.67	4.07	4.34	51.26	*P* = .005
				SCHNOS‐6	5	2.8	1.64	0.22	0.44	2.58	*P* = .026
Taha (2021)	LCSG	Primary	12	VAS‐function (left)	10	51.3	8.5	27.7	NR	23.6	*P* = .00
				VAS‐function (right)		51.2	5.31	27.2	NR	24	
				NOSE		80.1	NR	37.3	NR	42.8	*P* = .00
				SNOT‐22		1.98	NR	1.03	NR	0.95	*P* = .00
				NPIF (L/min)		111	NR	87	NR	24	*P* = .00
		Revision		VAS‐function (left)	16	44.94	23.59	42.5	NR	2.44	*P* = .00
				VAS‐function (right)		41.94	21.45	24.13	NR	17.81	*P* = .00
				NOSE		70	NR	38.75	NR	31.25	*P* = .00
				SNOT‐22		1.82	NR	1.02	NR	0.8	*P* = .00
				NPIF (L/min)		107.5	NR	99.37	NR	8.13	*P* = .00
Kondo (2020)	LCSG	Both	11	VAS‐function	69	58.93	24.16	40.44	NR	18.49	*P* < .001
				NOSE		53.75	27.5	14.25	NR	39.5	*P* < .001
				NPIF (L/min)		118.71	33.11	128.44	NR	9.73	*P* < .001
Ilhan (2015)	LCSG	Primary	12	NOSE	71	69.6	50.1	3.9	10.7	65.6	*P* = .001
Barham (2015)	LCSG	Revision	7	VAS‐function (left)	16	44.94	23.59	42.5	NR	2.44	*P* > .05
				VAS‐function (right)		41.94	21.45	24.13		17.81	*P* > .05
				NOSE		70	19	38.8	25.3	31.25	*P* > .05
				SNOT‐22		1.8	0.6	1	0.8	0.8	*P* > .05
				NPIF (L/min)		107.5	73.1	115.6	30.3	8.13	*P* > .05
Kofler (2023)	ABG	Primary	6	NOSE	20	15.4 [7‐20]*		5.4 [0‐16]*			*P* < .001
Maggon (2016)	ABG	Primary	6	NOSE	13	69.69	4.94	44.08	9.64	25.62	NR
				VAS‐function		69	4.9	43.5	2.1	24	NR
Bewick (2013)	ABG	Both	12	VAS‐function	27	73.5	NR	19	NR	54.5	*P* < .005
Sufyan (2013)	ABG	Both	12	NOSE	122	84	NR	15	NR	69	*P* < .001
Zoumalan (2012)	ABG	NR	9	Not comparable: rhinometry measurements only	14	NR	NR	NR	NR	NR	NR
Cervelli (2009)	ABG	Both	24	Not comparable: Likert scale only	80	NR	NR	NR	NR	NR	NR
Toriumi (1997)	ABG	NR	60	Not comparable: Likert scale only	46	NR	NR	NR	NR	NR	NR

Abbreviations: ABG, alar batten graft; FACE‐Q, Facial Aesthetic Clinical Evaluation‐Questionnaire; LCSG, lateral crural strut graft; NR, not recorded; NOSE, nasal obstruction symptom evaluation; NPIF, nasal peak inspiratory flow; SCHNOS, Standardized Cosmesis and Health Nasal Outcomes Survey; SNOT, Sino‐Nasal Outcomes Test; VAS, visual analog scale.

* = median.

### LCSG Cohort

A total of eight articles and 690 patients had documented postoperative outcomes following placement of LCSG for nasal valve incompetence.^12^, ^13^, ^14^, ^15^, ^16^, ^17^, ^18^, ^19^ The range in age of patients receiving LCSG was 18 to 52. Four articles included both primary and revision rhinoplasty cases.^12^, ^13^, ^16^, ^18^ All LCSGs were placed with an open rhinoplasty approach, but the origin of the LCSG varied, with two articles reporting the use of autologous costal cartilage, one article reporting the use of septal cartilage for all patients, and one article utilizing both septal and costal. Seven out of eight articles with outcomes following placement of LCSG reported patient scores on the NOSE both preoperatively and postoperatively. VAS scores were used by five out of eight of the articles, whereas the SCHNOS, SNOT‐22, and FACE‐Q were used least frequently. Four out of eight articles also reported objective outcomes using nasal peak inspiratory flow (NPIF) measurements.

The average time to follow‐up was 12.5 months. Of articles reporting NOSE scores, there was significant improvement in five out of six articles, with postoperative means ranging from 15.75 to 65.6 points of improvement following placement of the LCSG. One article reported median values as opposed to mean score, with statistically significant improvement in median NOSE score from 70 [55‐83] to 23 [12‐36] in the LCSG cohort.^17^ Of articles reporting functional VAS scores, all reported significant improvement, with postoperative means ranging 10.1 to 44.3 points of improvement. All articles, including SNOT‐22 and NPIF [8.1‐24.0] scores, reported significant improvement. A single article reported no significant improvement in any subjective outcome measurements 6.9 months after placement of the LCSG. Although functional gains were consistent, esthetic satisfaction was less uniformly measured. In the LCSG cohort, two studies employed the 10‐item SCHNOS‐C, reporting mean score improvements of 51.26 to 55.67 to points postoperatively. Those studies also used a visual analog scale for cosmesis (VAS‐C), with mean increases ranging from 3.5 to 4.9 points of improvement. Hismi et al incorporated the FACE‐Q esthetic module, demonstrating subscale improvements across nasal shape (50 [42‐69] to 74 [59‐93] points improvement), nostril symmetry (64 [34‐78] to 84 [64‐100] points improvement), and social confidence (73 [58‐81] to 80 [66‐96] points improvement).^17^ These validated patient‐reported outcome measures (PROMs) consistently showed statistically significant cosmetic enhancement after LCSG placement. Additionally, two studies reported SCHNOS‐6 values from the SCHNOS questionnaire, which assesses the shape of the nasal tip.^16^, ^19^ The range of improvement was 2.58 to 2.64 points, and this was found to be statistically significant.

### ABG Cohort

A total of seven articles and 362 patients had documented postoperative outcomes following placement of ABG for nasal valve incompetence.^7^, ^9^, ^20^, ^21^, ^22^, ^23^, ^24^ The range in age of patients receiving ABG was 18 to 75 years. Both primary and revision cases were included, and all ABGs were placed via the open rhinoplasty approach. Septal cartilage was the most frequently used graft type. Two articles reported subjective outcomes following ABG placement using a VAS scale. In addition, three articles reported subjective outcomes using the NOSE scale and three reported outcomes using either acoustic rhinometry or nonspecific Likert scale scores.

**Figure 2:**
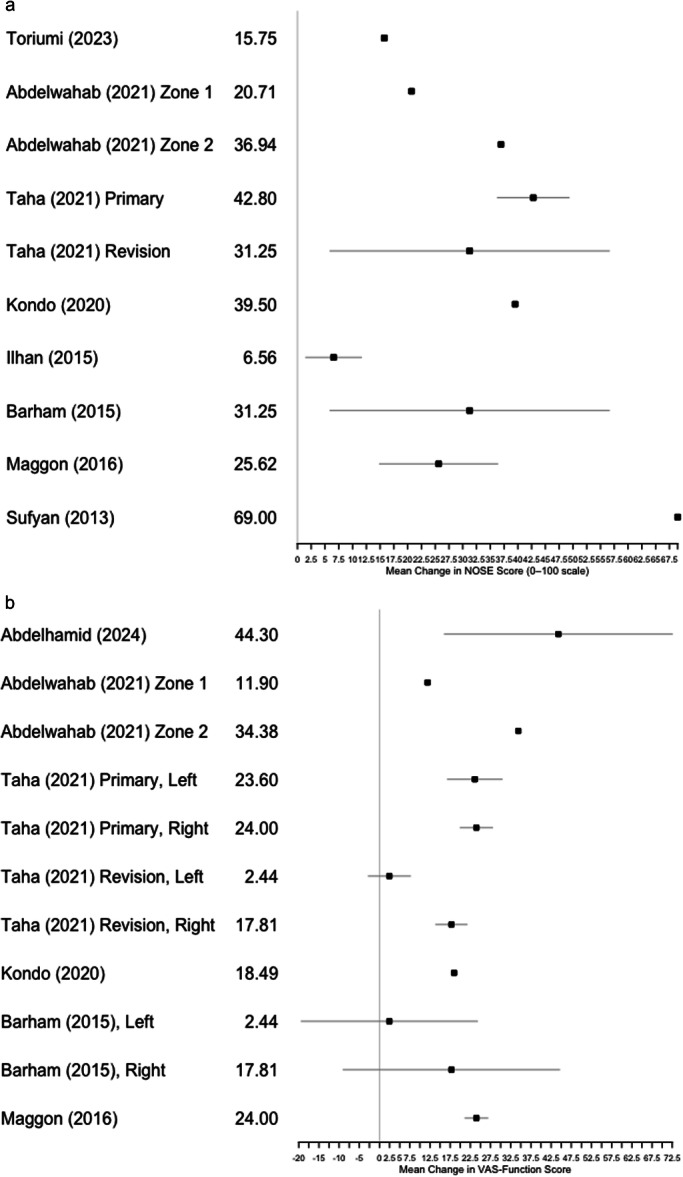
Descriptive forest plots of mean functional outcome improvements across studies. (A) Nasal obstruction symptom evaluation (NOSE) scores and (B) visual analog scale (VAS)‐function scores. Error bars represent ± 1 SD where available; studies without variance data are plotted as point estimates.

Average time to follow up was 21 (6‐60) months. Both NOSE and VAS‐F scores demonstrated statistically significant improvement, with postoperative means ranging from 25.6 to 69 and 24.0 to 54.5 points of improvement, respectively. All articles reporting subjective patient‐reported outcome measures documented a significant improvement following placement of the ABG. Rhinometry measurements were also significantly improved following placement of ABG for both articles reporting objective outcomes. None of the ABG articles utilized SNOT‐22 nor any cosmetic outcome measures like SCHNOS‐C, FACE‐Q, or VAS‐C. Comparative meta‐analysis of outcomes following placement of LCSG and ABG for treatment of nasal valve incompetence was unable to be performed due to significant heterogeneity between methods and results of selected references. Descriptive forest‐style plots of the mean NOSE and VAS‐function score improvements (post‐minus‐preoperative) were created, illustrating consistent functional benefit for both LCSG and ABG techniques (Figures 2A and B). Error bars represented ±1 SD where available, and studies without variance data are shown as point estimates.

## Discussion

This study aimed to systematically assess functional outcomes and postoperative cosmetic satisfaction rates for patients undergoing surgical placement of ABG or LCSG for nasal airway obstruction. Across the literature, both LCSG and ABG show significant improvement in multiple reported outcome metrics; however, the data for functional outcomes were significantly more robust than the cosmetic outcomes. The NOSE score was the most frequently used functional PROM for both surgical interventions—postoperative improvements ranged from 15.8 to 65.6 points after LCSG and 25.6 to 69 points after ABG. VAS‐function gains were similarly substantial (LCSG 10.1‐44.3 points, ABG 24.0‐54.5 points). These ranges highlight that, despite varied study designs and follow‐up durations (12.5 months on average for LCSG; 21 months for ABG), both techniques provide clinically meaningful relief of obstructive symptoms. However, due to a low number of included articles and significant heterogeneity between reported methods and outcomes, additional meta‐analysis was not able to be performed, and comparative conclusions cannot be drawn between the two graft types.

Previous literature has investigated the impact of functional rhinoplasty on patient‐reported outcomes. A recent systematic review and meta‐analysis of 16 studies concluded that functional rhinoplasty resulted in significant improvement in NOSE, SNOT‐22, and VAS scores in 971 patients.^25^ The impact of variables such as surgical technique and cartilage graft type on patient outcomes has not been explored. In 2022, Hismi et al compared patient‐reported outcomes among patients with nasal obstruction undergoing spreader grafts, LCSG, and alar rim grafts. All graft types resulted in a mean improvement in NOSE scores at the last postoperative visit, with no detectable difference between the analyzed groups.^17^ To our knowledge, our study is the first to attempt a systematic comparison of functional and cosmetic outcomes between LCSG and ABG specifically.

Of patients presenting to otolaryngologists with sinonasal complaints, more than 60% suffer from severe or extreme nasal obstruction based on NOSE score evaluation.^2^ Previous literature has associated nasal valve collapse with significant functional deficiencies resulting in a large reduction in patient quality‐of‐life scores.^26^ LCSG and ABG are just two of several tools available to the rhinoplasty surgeon for addressing nasal valve insufficiency and improving patient quality of life, although they differ in the degree of surgical dissection needed for placement. When considering the differential anatomical placement of these grafts, there may be functional and esthetic differences between them, with the expectation that ABG are more visible but potentially with less space consumed within the airway. However, extrapolation of data across multiple studies was not possible to allow for granular comparison. Moreso, heterogeneity in the size of grafts or technical nuances makes comparison difficult. Nonetheless, data suggest that both LCSG and ABG appear to have a positive impact on patient quality of life and surgical outcomes. Therefore, either graft may be a viable option for the large population of patients experiencing nasal obstruction.

Recent clinical practice guidelines have advocated for the use of PROMs to gauge obstructive symptoms and the success of surgical intervention following both functional and cosmetic rhinoplasty.^27^, ^28^ The most frequently used patient‐reported outcome measures found in our selected references were the NOSE and VAS scales. The NOSE score is the most frequently used patient‐reported outcome measure in rhinoplasty literature.^28^ This validated scale has consistently been a reliable and sensitive evaluation of nasal obstruction in this patient population.^29^ However, a thorough understanding of the limitations of this scale is warranted.

Functional rhinoplasty intervention may lead to significant modifications to nasal appearance. In addition, cosmetic rhinoplasty may also have an impact on the nasal valve and nasal breathing. Due to the complex interplay of functional and cosmetic outcomes, a PROM that incorporates both obstruction and esthetic concerns is more appropriate for the measurement of quality of life following rhinoplasty interventions. Outcome measures such as the SCHNOS, which incorporate both functional and cosmetic measures while also quantifying positive effects, are better options for gauging the overall success of surgical intervention.^30^ SCHNOS‐C, VAS‐C, and FACE‐Q data in the LCSG cohort demonstrated statistically significant esthetic benefits. By contrast, none of the ABG studies utilized SCHNOS‐C, VAS‐C, or FACE‐Q; instead, cosmetic outcomes were assessed via nonstandard Likert scales or surgeon‐graded rhinometry, which generally reported subjective improvement but preclude direct comparison. Toriumi et al did evaluate esthetic outcomes with an internal Likert scale of 1 (deformity) to 5 (excellent). They note that the mean esthetic score for all patients was 4 on a scale of 5, indicating improvement in esthetic appearance. Similarly, Cervelli et al evaluated esthetic results with a VAS scale that was completed by both the surgeon and patient. The patient's score was the same or better than the surgeon for all participants. A lack of objective outcome measures and standardized PROMs across the board limits conclusions that may be drawn from currently available rhinoplasty literature and prevents comparison to ABG. Standardization of outcome analyses and consistent use of validated measures will better allow for extrapolation and comparison of data across studies. Validated physician‐graded systems also exist for nasal valve collapse and would be better able to elucidate the effects of surgical techniques aimed at correcting dynamic and static sidewall issues, such as the LCSG and ABG.^31^, ^32^

Our study has several limitations to consider. Despite a thorough systematic review search, a limited number of articles met the criteria for inclusion. Articles were most frequently excluded due to incorrect cartilage graft type and lack of comparable outcomes. In addition, as previously stated, reported outcomes across rhinoplasty literature vary widely both in selection and method of use. This significantly limits the extrapolation and analysis of similar data across studies. Because of this, no strong or comparative conclusions can be drawn between the two graft types. Large, prospective, and multi‐institutional studies investigating outcome differences between graft types using validated cosmetic and functional patient‐reported outcome measures and physician‐graded systems are needed to better understand differences in surgical success between these two techniques.

## Conclusion

This study aimed to systematically assess currently available functional outcomes and postoperative cosmetic satisfaction rates for patients undergoing surgical placement of ABG or LCSG for nasal airway obstruction due to nasal valve incompetence. Both LCSG and ABG placement resulted in significant improvement across multiple functional outcome metrics. Cosmetic satisfaction appeared favorable but remains undercharacterized in comparison to functional outcomes. There was significant heterogeneity between study methods and results, limiting strong conclusions about the comparison of these two graft types. Prospective outcome studies between the graft types are needed for further analysis.

## Author Contributions

**Jarrett M. Jackson**: Conceptualization; methodology; data collection; manuscript writing. **Nicole G. DeSisto**: Conceptualization; methodology; data collection; manuscript writing. **Rahul K. Sharma**: Methodology; statistical analysis; manuscript writing. **Rachel Walden**: Methodology; statistical analysis. **Shiayin F. Yang**: Supervision; review and editing. **Scott J. Stephan**: Supervision; review and editing. **Priyesh N. Patel**: Study design; supervision; project administration; review and editing.

## Disclosures

### Competing interests

The authors declare no conflicts of interest.

### Funding source

Funding from Vanderbilt University Medical Center, Department of Otolaryngology.

## Supporting information


**Supporting Information**.

**Supporting Information**.

## References

[1] Hsu DW , Suh JD . Anatomy and physiology of nasal obstruction. Otolaryngol Clin North Am. 2018;51(5):853‐865. 10.1016/j.otc.2018.05.001 29941182

[2] Clark DW , Del Signore AG , Raithatha R , Senior BA . Nasal airway obstruction: prevalence and anatomic contributors. Ear Nose Throat J. 2018;97(6):173‐176. 10.1177/014556131809700615 30036414

[3] Chandra RK , Patadia MO , Raviv J . Diagnosis of nasal airway obstruction. Otolaryngol Clin North Am. 2009;42(2):207‐225. 10.1016/j.otc.2009.01.004 19328887

[4] Avashia YJ , Glener AD , Marcus JR . Functional nasal surgery. Plast Reconstr Surg. 2022;150(2):439. 10.1097/prs.0000000000009290 35895523

[5] Hamilton GS . The external nasal valve. Facial Plast Surg Clin North Am. 2017;25(2):179‐194. 10.1016/j.fsc.2016.12.010 28340649

[6] Ji KSY , Krane NA . Surgical treatment of dynamic nasal collapse. Facial Plast Surg. 2022;38(04):339‐346. 10.1055/a-1825-2610 35419774

[7] Toriumi DM , Josen J , Weinberger M , Tardy ME . Use of alar batten grafts for correction of nasal valve collapse. Arch Otolaryngol Head Neck Surg. 1997;123(8):802‐808. 10.1001/archotol.1997.01900080034002 9260543

[8] Goudakos JK , Fishman JM , Patel K . A systematic review of the surgical techniques for the treatment of internal nasal valve collapse: where do we stand? Clin Otolaryngol. 2017;42(1):60‐70. 10.1111/coa.12664 27119792

[9] Cervelli V , Spallone D , Bottini JD , et al. Alar batten cartilage graft: treatment of internal and external nasal valve collapse. Aesthetic Plast Surg. 2009;33(4):625‐634. 10.1007/s00266-009-9349-5 19421808

[10] Millman B . Alar batten grafting for management of the collapsed nasal valve. Laryngoscope. 2002;112(3):574‐579. 10.1097/00005537-200203000-00030 12148874

[11] Gunter JP , Friedman RM . Lateral crural strut graft: technique and clinical applications in rhinoplasty. Plast Reconstr Surg. 1997;99(4):943‐952. 10.1097/00006534-199704000-00001 9091939

[12] Taha MA , Hall CA , Zylicz HE , et al. Costal cartilage lateral crural strut graft for correction of external nasal valve dysfunction in primary and revision rhinoplasty. Ear Nose Throat J. 2023;102(3):175‐180. 10.1177/0145561320983940 33559494

[13] Kondo M , Orgain C , Alvarado R , Marcells GN , Harvey RJ . The effects of lateral crural tensioning with an articulated alar rim graft versus lateral crural strut graft on nasal function. Facial Plast Surg Aesthet Med. 2020;22(4):281‐285. 10.1089/fpsam.2020.0056 32326747

[14] Ilhan AE , Saribas B , Caypinar B . Aesthetic and functional results of lateral crural repositioning. JAMA Facial Plast Surg. 2015;17(4):286‐292. 10.1001/jamafacial.2015.0590 26086322

[15] Barham HP , Knisely A , Christensen J , Sacks R , Marcells GN , Harvey RJ . Costal cartilage lateral crural strut graft vs cephalic crural turn‐in for correction of external valve dysfunction. JAMA Facial Plast Surg. 2015;17(5):340‐345. 10.1001/jamafacial.2015.0925 26247619

[16] Abdelwahab M , Patel P , Kandathil CK , Wadhwa H , Most SP . Effect of lateral crural procedures on nasal wall stability and tip aesthetics in rhinoplasty. Laryngoscope. 2021;131(6):E1830‐E1837. 10.1002/lary.29389 33459395

[17] Hismi A , Burks CA , Locascio JJ , Lindsay RW . Comparative effectiveness of cartilage grafts in functional rhinoplasty for nasal sidewall collapse. Facial Plast Surg Aesthet Med. 2022;24(3):240‐246. 10.1089/fpsam.2021.0219 34494891

[18] Toriumi DM , Cristel RT . Lateral crural repositioning: implications on nasal function. Facial Plast Surg. 2023;39(05):547‐555. 10.1055/s-0043-1771499 37709290

[19] Abdelhamid AS , Kimura KS , El Abany A , Kandathil CK , Most SP . Patient outcomes in lateral crural repositioning and reconstruction in revision rhinoplasty. Facial Plast Surg Aesthet Med. 2024;26(1):9‐14. 10.1089/fpsam.2022.0434 37115534

[20] Zoumalan RA , Morris LGT , Zeitler DM , Shah AR . Effects of various submucous resection techniques of septal cartilage on nasal tip projection. Int Forum Allergy Rhinol. 2011;1(1):78‐82. 10.1002/alr.20009 22287312

[21] Sufyan AS , Hrisomalos E , Kokoska MS , Shipchandler TZ . The effects of alar batten grafts on nasal airway obstruction and nasal steroid use in patients with nasal valve collapse and nasal allergic symptoms: a prospective study. JAMA Facial Plast Surg. 2013;15(3):182‐186. 10.1001/jamafacial.2013.974 23450346

[22] Bewick JC , Buchanan MA , Frosh AC . Internal nasal valve incompetence is effectively treated using batten graft functional rhinoplasty. Int J Otolaryngol. 2013;2013:1‐5. 10.1155/2013/734795 PMC3638702 23653651

[23] Das A . Posttraumatic nasal valve collapse: is alar batten graft the answer? Int J Clin Rhinol. 2016;9(3):125‐129. 10.5005/jp-journals-10013-1285

[24] Department of Otorhinolaryngology, Medical University Innsbruck, Innsbruck, Austria , Kofler B , Böpple T , Runge A , Völklein C . Batten grafts in patients with valve stenosis—functional outcome. B‐ENT. 2024;19(1):32‐37. 10.5152/b-ent.2023.22321

[25] Chen K , Zhou L . The effect of functional rhinoplasty on quality of life: a systematic review and meta‐analysis. Aesthetic Plast Surg. 2024;48(5):847‐854. 10.1007/s00266-023-03390-3 37173413

[26] Samra S , Steitz JT , Hajnas N , Toriumi DM . Surgical management of nasal valve collapse. Otolaryngol Clin North Am. 2018;51(5):929‐944. 10.1016/j.otc.2018.05.009 30017094

[27] Ishii LE , Tollefson TT , Basura GJ , et al. Clinical practice guideline: improving nasal form and function after rhinoplasty executive summary. Otolaryngol Head Neck Surg. 2017;156(2):205‐219. 10.1177/0194599816683156 28145848

[28] Rossi Meyer MK , Most SP . Quantifying the subjective experience of nasal obstruction: a review. Facial Plast Surg. 2024;40(03):336‐340. 10.1055/a-2160-4998 37625460

[29] Stewart MG , Witsell DL , Smith TL , Weaver EM , Yueh B , Hannley MT . Development and validation of the nasal obstruction symptom evaluation (NOSE) scale. Otolaryngol Head Neck Surg. 2004;130(2):157‐163. 10.1016/j.otohns.2003.09.016 14990910

[30] Moubayed SP , Ioannidis JPA , Saltychev M , Most SP . The 10‐item Standardized Cosmesis and Health Nasal Outcomes Survey (SCHNOS) for functional and cosmetic rhinoplasty. JAMA Facial Plast Surg. 2018;20(1):37‐42. 10.1001/jamafacial.2017.1083 28880988 PMC5833673

[31] Tsao GJ , Fijalkowski N , Most SP . Validation of a grading system for lateral nasal wall insufficiency. Allergy Rhinol (Providence). 2013;4(2):E66‐E68. 10.2500/ar.2013.4.0054 24124639 PMC3793115

[32] Patel B , Virk JS , Randhawa PS , Andrews PJ . The internal nasal valve: a validated grading system and operative guide. Eur Arch Otrhinolaryngol. 2018;275(11):2739‐2744. 10.1007/s00405-018-5142-x PMC6208712 30293091

