# Supplementary material for: Alar Battens Grafts Versus Lateral Crural Strut Grafts: A Systematic Review of Postoperative Outcomes
Source: Otolaryngol Head Neck Surg. 2025 Sep 8;173(6):1328–36. doi: 10.1002/ohn.70010 (PMC12661469; doi:10.1002/ohn.70010)
Supplement: Supplementary file 1 — Supporting Information. [file OHN-173-1328-s001.docx]

Supplemental 1: Search Algorithm

**Description:** Search algorithm. A document depicting the systematic search algorithm and Boolean operators used to query each database.

*Initial Search (Conducted October 2022)*

*Date Range: January 1995 – October 1 2022*

**Pub-Med Search:**

(block* OR obstruct* OR collaps* OR incompeten* OR "Nasal Obstruction"[Mesh]) AND (Lateral Crura[tiab] OR lateral crural strut graft*[tiab] OR Batten graft*[tiab] OR alar batten*[tiab] OR alar strut graft*[tiab])

Results: 109

("Nose"[Mesh] OR nose OR nasal OR rhinoplasty OR Rhinoplasties OR "Rhinoplasty"[Mesh]) AND (Lateral Crura[tiab] OR lateral crural strut graft*[tiab] OR Batten graft*[tiab] OR alar batten*[tiab] OR alar strut graft*[tiab])

Results: 277

**(Lateral Crura[tiab] OR lateral crural strut graft*[tiab] OR Batten graft*[tiab] OR alar batten*[tiab] OR alar strut graft*[tiab])**

**Results: 280**

**Total added to EndNote: 666**

**Duplicates removed: 386**

**Remaining: 280**

**Embase Search:**

(exp nose obstruction/ or (block* or obstruct* or collaps* or incompeten*).mp.) and (lateral crura* or lateral crural strut graft* or batten graft* or alar batten* or alar strut graft*).mp.

Results: 137

(exp nose/ or exp rhinoplasty/ or (nose or nasal or rhinoplasty or rhinoplasties).mp.) and (lateral crura* or lateral crural strut graft* or batten graft* or alar batten* or alar strut graft*).mp.

Results: 381

(lateral crura* or lateral crural strut graft* or batten graft* or alar batten* or alar strut graft*).mp.

Results: 401

Total added to EndNote: 919

Duplicates removed: 511

Remaining: 408

Total searches combined: 688

Duplicates removed: 208

**FINAL TOTAL: 480**

Embase: 200

PubMed: 280

Added to Covidence: 480

1 additional duplicate removed

Total: 479

*Updated Search (Conducted May 2025)*

*Date Range: October 1 2022 – May 2025*

**PubMed Search:**

(((block* OR obstruct* OR collaps* OR incompeten* OR "Nasal Obstruction"[Mesh]) AND (Lateral Crura[tiab] OR lateral crural strut graft*[tiab] OR Batten graft*[tiab] OR alar batten*[tiab] OR alar strut graft*[tiab])) AND ("cosmetic outcome"[tiab] OR "aesthetic outcome"[tiab] OR outcome*[tiab])) AND (("2022/10/01"[Date - Publication] : "2025/05/16"[Date - Publication]))

Results: 7

(("Nose"[Mesh] OR nose OR nasal OR rhinoplasty OR Rhinoplasties OR "Rhinoplasty"[Mesh]) AND

(Lateral Crura[tiab] OR lateral crural strut graft*[tiab] OR Batten graft*[tiab] OR alar batten*[tiab] OR alar strut graft*[tiab]) AND

("cosmetic outcome"[tiab] OR "aesthetic outcome"[tiab] OR outcome*[tiab]))

AND ("2022/10/01"[Date - Publication] : "2025/05/16"[Date - Publication])

Results: 20

((Lateral Crura[tiab] OR lateral crural strut graft*[tiab] OR Batten graft*[tiab] OR alar batten*[tiab] OR alar strut graft*[tiab]) AND

("cosmetic outcome"[tiab] OR "aesthetic outcome"[tiab] OR outcome*[tiab]))

AND ("2022/10/01"[Date - Publication] : "2025/05/16"[Date - Publication])

Results: 20

**Total added to EndNote: 47**

**Duplicates removed: 27**

**Remaining: 20**

**Embase Search**

('nose obstruction'/exp OR block*:ab,ti OR obstruct*:ab,ti OR collaps*:ab,ti OR incompeten*:ab,ti) AND

(lateral crura*:ab,ti OR 'lateral crural strut graft*':ab,ti OR 'batten graft*':ab,ti OR 'alar batten*':ab,ti OR 'alar strut graft*':ab,ti) AND

('cosmetic outcome*':ab,ti OR 'aesthetic outcome*':ab,ti OR outcome*:ab,ti) AND

[2022-2025]/py

Results: 12

('nose'/exp OR 'rhinoplasty'/exp OR nose:ab,ti OR nasal:ab,ti OR rhinoplasty:ab,ti OR rhinoplasties:ab,ti) AND

(lateral crura*:ab,ti OR 'lateral crural strut graft*':ab,ti OR 'batten graft*':ab,ti OR 'alar batten*':ab,ti OR 'alar strut graft*':ab,ti) AND

('cosmetic outcome*':ab,ti OR 'aesthetic outcome*':ab,ti OR outcome*:ab,ti) AND

[2022-2025]/py

Results: 27

(lateral crura*:ab,ti OR 'lateral crural strut graft*':ab,ti OR 'batten graft*':ab,ti OR 'alar batten*':ab,ti OR 'alar strut graft*':ab,ti) AND

('cosmetic outcome*':ab,ti OR 'aesthetic outcome*':ab,ti OR outcome*:ab,ti) AND

[2022-2025]/py

Results: 35

Total added to Endnote: 74

Duplicates Removed: 41

Remaining: 33

Total searches combined: 53

Duplicates removed: 1

**FINAL TOTAL: 38**

Embase: 18

PubMed: 20

Added to Covidence: 38

Total: 38
