# Supplementary material for: Alar Battens Grafts Versus Lateral Crural Strut Grafts: A Systematic Review of Postoperative Outcomes
Source: Otolaryngol Head Neck Surg. 2025 Sep 8;173(6):1328–36. doi: 10.1002/ohn.70010 (PMC12661469; doi:10.1002/ohn.70010)
Supplement: Supplementary file 2 — Supporting Information. [file OHN-173-1328-s002.docx]

Supplement 2: Newcastle-Ottowa Scores

| **Study** | **Selection (0–4)** | **Comparability**  **(0–2)** | **Outcome**  **(0–3)** | **Total (0–9)** |
| --- | --- | --- | --- | --- |
| Toriumi (1997) | 2 | 1 | 3 | 6 |
| Zoumalan (2012) | 4 | 2 | 3 | 9 |
| Cervelli (2009) | 3 | 2 | 3 | 8 |
| Bewick (2013) | 4 | 2 | 3 | 9 |
| Sufyan (2013) | 3 | 2 | 3 | 8 |
| Maggon (2016) | 2 | 2 | 3 | 7 |
| Taha (2021) | 4 | 2 | 3 | 9 |
| Kondo (2020) | 4 | 2 | 3 | 9 |
| Ilhan (2015) | 3 | 2 | 3 | 8 |
| Barham (2015) | 4 | 2 | 3 | 9 |
| Abdelwahab (2021) | 4 | 2 | 3 | 9 |
| Hismi (2022) | 4 | 3 | 3 | 9 |
| Kofler (2023) | 3 | 2 | 2 | 7 |
| Toriumi (2023) | 4 | 2 | 3 | 9 |
| Abdelhamid (2024) | 4 | 2 | 3 | 9 |
